# Fumigant Antifungal Activity of Myrtaceae Essential Oils and Constituents from *Leptospermum petersonii* against Three *Aspergillus* Species

**DOI:** 10.3390/molecules170910459

**Published:** 2012-09-03

**Authors:** Eunae Kim, Il-Kwon Park

**Affiliations:** Division of Forest Insect Pests and Diseases, Korea Forest Research Institute, Seoul 130-712, Korea; Email: kea7013@gmail.com

**Keywords:** antifungal activity, Myrtaceae essential oil, *Leptospermum petersonii*, *Aspergillus ochraceus*, *Aspergillus flavus*, *Aspergillus niger*

## Abstract

Commercial plant essential oils obtained from 11 Myrtaceae plant species were tested for their fumigant antifungal activity against *Aspergillus ochraceus*, *A. flavus*, and *A. niger*. Essential oils extracted from * Leptospermum* *petersonii* at air concentrations of 56 × 10^−3^ mg/mL and 28 × 10^−3^ mg/mL completely inhibited the growth of the three *Aspergillus* species. However, at an air concentration of 14 × 10^−3^ mg/mL, inhibition rates of *L. petersonii* essential oils were reduced to 20.2% and 18.8% in the case of *A. flavus* and *A. niger*, respectively. The other Myrtaceae essential oils (56 × 10^−3^ mg/mL) only weakly inhibited the fungi or had no detectable affect. Gas chromatography-mass spectrometry analysis identified 16 compounds in *L. petersonii* essential oil. The antifungal activity of the identified compounds was tested individually by using standard or synthesized compounds. Of these, neral and geranial inhibited growth by 100%, at an air concentration of 56 × 10^−3^ mg/mL, whereas the activity of citronellol was somewhat lover (80%). The other compounds exhibited only moderate or weak antifungal activity. The antifungal activities of blends of constituents identified in *L. petersonii* oil indicated that neral and geranial were the major contributors to the fumigant and antifungal activities.

## 1. Introduction

*Aspergillus* species are not only responsible for the spoilage of food and feed [1], but they also produce several toxins. Aflatoxins and ochratoxin A (OTA) are secondary metabolites produced by *Aspergillus* species, such as *A. flavus*, *A. parasiticus*, *A. nomius*, and *A. ochraceus* [2]. Aflatoxins are potent carcinogens for animals and humans [3]. OTA induces nephrotoxic, immunotoxic, teratogenic, and carcinogenic effects [4]. Plant essential oils are good candidates for the control of *Aspergillus* species, because they consist of many bioactive chemicals. Further, because the components of plant essential oils are highly volatile, they do not persist when used in fields or in water [5,6]. Many plant essential oils and their individual components have been reported to demonstrate antifungal activities against *Aspergillus* species [7,8,9,10,11,12,13]. The family Myrtaceae contains at least 3,000 species within 130–150 genera, and it is distributed in several tropical and warm-temperature areas. Many essential oils produced by these species exhibit insecticidal, antifungal, or nematicidal activity [6,14,15,16]. The pesticidal activity of *Eucalyptus* essential oils have been greatly reviewed by Batish *et al*. [17]. Furthermore, essential oils from Myrtaceae have multiple uses, *i.e.*, in perfumery, pharmaceutical, and in other industries [18]. Because of their many advantages described above, we assessed the fumigant antifungal activities of 11 Myrtaceae plant essential oils and their components against three *Aspergillus* fungi species with the goal of discovering new and safe alternatives for controlling the growth of *Aspergillus* species. 

## 2. Results and Discussion

### 2.1. Fumigant Antifungal Activity of Plant Essential Oils

The abilities of 11 Myrtaceae essential oils to inhibit the growth of three *Aspergillus* species are shown in Table 1. The essential oils produced by *Leptospermum petersonii* inhibited growth by 100% at air concentrations of 56 × 10^−3^ mg/mL and 28 × 10^−3^ mg/mL. However, at an air concentration of 14 × 10^−3^ mg/mL, inhibition rates of *L. petersonii* essential oils were reduced to 20.2% and 18.8% against *A. flavus* and *A. niger*, respectively. Inhibition rates of essential oils extracted from *Eucalyptus globulus*, *Melaleuca dissitiflora*, and *M. quinquenervia* at an air concentration of 56 × 10^−3^ mg/mL against *A. ochraceus* were 26.1, 21.5, and 26.5%, respectively, and essential oils from *M. dissitiflora* and *M. quinquenervia* inhibited the growth of *A. flavus* by 22.9% and 34.45%, respectively at the same air concentration. None of the other Myrtaceae essential oils showed antifungal activity against either of the three *Aspergillus* species at an air concentration of 56 × 10^−3^ mg/mL. In the study, only *L. petersonii* essential oils demonstrated strong antifungal activities against the three *Aspergillus* species. The antifungal activities of Myrtaceae plant essential oils [19,20,21,22], including those of *L. petersonii* essential oils and constituents have been reported previously [23,24]. Park *et al*. [25] reported that essential oils and major constituents extracted from *L. petersonii* such as citronellal, neral, and geranial show antifungal activity against various dermatophytes, such as *Microsporum canis*, *Trichophyton mentagrophytes*, and *Microsporum gypseum.*

**Table 1:** Fumigant antifungal activities of 11 Myrtaceae essential oils against three *Aspergillus* species.

Plant species	Conc., ×10^−3^ mg/mL air	Inhibition rate (Mean ± S.E., %)		
		*Aspergillus ochraceus*	*Aspergillus flavus*	*Aspergillus niger*
*Eucalyptus citriodora*	56	0d ^1^	23.5 ± 2.6c	0c
*Eucalyptus polybractea*	56	0d	0d	0c
*Eucalyptus smithii*	56	0d	0d	0c
*Eucalyptus radiata*	56	0d	0d	0c
*Eucalyptus dives*	56	0d	0d	0c
*Eucalyptus globulus*	56	26.1 ± 0.4b	0d	0c
*Melaleuca dissitiflora*	56	21.5 ± 0.6c	22.9 ± 1.9c	0c
*Melaleuca quinquenervia*	56	26.5 ± 0.4b	34.4 ± 7.0b	0c
*Melaleuca uncinata*	56	0d	0d	0c
*Melaleuca linariifolia*	56	0d	0d	0c
*Leptospermum petersonii*	56	100a	100a	100a
	28	100a	100a	100a
	14	0d	20.2 ± 4.3c	18.8 ± 13.4b
		F_13,42_ = 0.25	F_13,42_ = 5.68	F_13,42_ = 12.84
		*p* < 0.0001	*p* < 0.0001	*p* < 0.0001

^1^ Mean values within a column followed by same letters are not significantly different (Scheffe’s test).

### 2.2. Chemical Components of Plant Essential Oils

The chemical components of *L. petersonii* are described in our previous study [15] and are listed in Table 2. Geranial (29.91%) and neral (22.83%) were the major constituents of *L. petersonii* oils, followed by citronellal (16.72%), isopulegol (5.20%), and linalool (3.25%). Seasonal variation in the chemical composition of *Leptospermum* oils has been described by Demuner *et al*. [26]. They report that geranial, neral, and citronellal were the major components of *L. petersonii* grown in Brazil, similar to our result. They also reported the difference in abundance of each chemical according to harvest season. The abundances of neral and geranial were higher during rainy season than those during dry season. This indicates that the essential oil of *L. petersonii* harvested in rainy season is a better source for isolating antifungals.

The chemical compositions of other *Eucalyptus* oils are well characterized [6,15,27]. The main components of *Eucalyptus* oils tested in our study were 1,8-cineole, citronellal, *α*-pinene, *p*-cymene, limonene, and *α*-terpineol, none of which exhibited detectable antifungal activity. This explains why essential oils extracted from *Eucalyptus* species do not show antifungal activity against *Aspergillus* species. The chemical compositions of *Melaleuca quinquenerivia*, *M. dissitiflora*, *M. linariifolia*, and *M. uncinata* oils have been determined by Lee *et al*. [15] and Yeom [27], and the major constituents include terpinen-4-ol, 1,8-cineole, limonene, *E*-nerolidol, isopulegol, *α*-terpineol, linalool, and *p*-cymene. Most of these compounds did not show antifungal activity against *Aspergillus* species in this study. Chemical composition of essential oils extracted from *Eucalyptus* and *Melaleuca* species explain the reason for the weak or absence of antifungal activity against *Aspergillus* species in this study. Essential oils such as those isolated from *Melissa officinalis*, *Lippia alba*, *Litsea cubeba*, and *Cymbopogon citratus* contain neral and geranial as main components; therefore, we considered them as good candidates for controlling *Aspergillus* species [28,29,30,31].

**Table 2:** Chemical composition of *Leptospermum petersonii* essential oil ^1^.

No.	Compounds	RI ^2^		Amount (w/w, %)
		DB-1	FFAP	
1	*α*-Pinene	928	1014	0.54
2	6-Methyl-5-heptene-2-one	964	1330	0.77
3	*β*-Pinene	967	1098	0.24
4	*β*-Myrcene	981	1155	1.26
*5*	*p*-Cymene	1012	1261	0.07
6	1,8-Cineole	1018	1196	0.09
7	Limonene	1020	1189	0.11
8	Linalool	1084	1536	3.25
9	Isopulegol	1128	1559	5.20
10	Citronellal	1131	1470	16.72
11	Citronellol	1211	1753	6.00
12	Neral	1216	1670	22.83
13	Geraniol	1236	1834	3.63
14	Geranial	1245	1721	29.91
15	*β*-Caryophyllene	1414	1583	0.14
16	Aromadendrene	1435	1597	0.08
	Sum of identified compounds			90.85

^1^ Chemical components of *L. peterisonii* have already been reported in our previous study [15]; ^2^ Retention indices of plant essential oils; Components were identified by co-injection with authentic standard on 2 columns.

### 2.3. Antifungal Activities of Individual Compounds

The fumigant antifungal activities of compounds identified in *L. petersonii* are shown in Table 3. The activity varied per the test compounds and the dose. The growth of *A. ochraceus*, was completely inhibited by neral and geranial at air concentrations of 56 × 10^−3^ and 28 × 10^−3^ mg/mL. The fumigant antifungal activity of neral remained at 100% at an air concentration of 14 × 10^−3^ mg/mL, but was reduced to 34.1% at 7 × 10^−3^ mg/mL. In contrast, the inhibition rate of geranial at an air concentration of 14 × 10^−3^ mg/mL was 20.1%. The inhibition rate of citronellol against *A. ochraceus* was 83.7% at an air concentration of 56 × 10^−3^ mg/mL, but was reduced to 29.4% at an air concentration of 28 × 10^−3^ mg/mL. Geraniol inhibited the growth of *A. ochraceus* by 64.6% and 38.4% at air concentration of 56 × 10^−3^ and 28 × 10^−3^ mg/mL, respectively. The other compounds tested were inhibitory at rates less than 50%.

**Table 3:** Fumigant antifungal activity of compounds identified in *Leptospermum petersonii* essential oils against three *Aspergillus* species.

Compounds	Conc., ×10^−3^ mg/mL air	Inhibition rate (Mean±S.E., %)		
		*Aspergillus ochraceus*	*Aspergillus flavus*	*Aspergillus niger*
*α*-Pinene	56	0f ^1^	0d	0e
6-Methyl-5-heptene-2-one	56	0f	0d	0e
*β*-Pinene	56	0f	0d	0e
*β*-Myrcene	56	0f	0d	0e
*p*-Cymene	56	0f	0d	0e
1,8-Cineole	56	0f	0d	0e
Limonene	56	0f	0d	0e
Linalool	56	36.6 ± 0.8d	0d	0e
Isopulegol	56	34.6 ± 3.2d	0d	33.4 ± 2.2cd
Citronellal	56	0f	0d	16.2 ± 11.8de
Citronellol	56	83.7 ± 3.3b	90 ± 0.7a	81.1 ± 9.0b
	28	29.4 ± 3.0de	37.7 ± 4.1b	35.3 ± 7.0c
Neral	56	100a	100a	100a
	28	100a	26.9 ± 2.1bc	0e
	14	100a	-^2^	-
	7	34.1 ± 4.2d	-	-
Geraniol	56	64.6 ± 7.5c	36.7 ± 11.3b	77.1 ± 9.8b
	28	38.4 ± 4.4d	-	0e
Geranial	56	100a	100a	100a
	28	100a	29.8±3.3bc	0e
	14	20.1 ± 3.8e	22.3 ± 3.5c	-
*β*-Caryophyllene	56	0f	0d	0e
Aromadendrene	56	0f	0d	0e
		F_22,69_ = 6.14	F_19,60_ = 8.77	F_19,60 _= 18.71
		*p* < 0.0001	*p* < 0.0001	*p* < 0.0001

^1^ Means values within a column followed by same letters are not significantly different (Sheffe’s test); ^2^ Not tested.

Neral, geranial, and citronellol inhibited the growth of *A. flavus* by >90% at an air concentration of 56 × 10^−3^ mg/mL, but only reached 40% at 28 × 10^−3^ mg/mL. Other compounds showed only weak or no inhibition against *A. flavus*. Neral, geranial, citronellol, and geraniol inhibited the growth of *A. niger* by 100%, 100%, 81.1%, and 77.1%, respectively, at concentration of 56 × 10^−3^ mg/mL, whereas only weak or no inhibition was at a concentration of 28 × 10^−3^ mg/mL. Other compounds exhibited either weak or no antifungal activity against *A. niger* at an air concentration of 56 × 10^−3^ mg/mL. Lee *et al*. [15] and Park *et al*. [25] have reported the bioactivity of geranial and neral compounds against phytopathogens and dermatophytes. Shukla *et al*. [32] also reported the antifungal activity of neral and geranial against *Aspergillus flavus*. Citronellol [33] along with citral, citronellal, citronellol, and geraniol also inhibits the growth of *A. flavus*. The findings of Setzer *et al*. [34] that a mixture of neral and geranial potently inhibits the growth of *A. niger* agrees with the results of our present study, except that we were unable to demonstrate that citronellal was inhibitory. Furthermore, citronellol and geraniol were potent inhibitors of the growth of *A. niger*. The difference might be attributed to differences in the bioassay methods used. Here, we used a fumigant assay, not one that involves more direct contact as in the studies cited.

Structure-activity relationships of certain phytochemicals obtained from plant essential oils against fungi have been well studied. Antifungal activities of unsaturated aldehydes (citral, cinnamic aldehyde, and citronellal) and an unsaturated alcohol (geraniol) are stronger than those of hydrocarbons, such as camphene, limonene, and α-terpinene against *A. niger*, *Fusarium oxysporum*, and *Penicillium digitatum* [35]. The studies of Lee *et al*. [15] demonstrate that aldehydes and alcohols are generally more toxic than hydrocarbons for three plant pathogens, namely, *Phytophthora cactorum*, *Cryphonectria parasitica*, and *Fusarium circinatum*. The results of our present study are consistent with their findings. Thus, aldehydes (neral, geranial) and alcohols (citronellol, geraniol) were more effective antifungals against the three *Aspergillus* species than were hydrocarbons, such as *α*-pinene, 6-methyl-5-heptene-2-one, *β*-pinene, *β*-myrcene, *p*-cymene, limonene, and *β*-caryophyllene. Further, there were significant differences in the antifungal activities among chemicals possessing the same functional group. For example, inhibitory activities of primary alcohol groups, such as present in citronellol and geraniol, were greater than those of secondary (isopulegol) and tertiary alcohols (linalool). This result indicates that the position of the hydroxyl group is related to the antifungal activity and is consistent with the results of our previous research [15]. For aldehydes, the antifungal activity of neral and geranial was very strong, but citronellal demonstrated either very weak or no detectable inhibition of the growth of the three *Aspergillus* species. The studies of Moleyar and Narasimham [35] and Lee *et al*. [15] demonstrate that *α,β*-unsaturated carbonyl compounds, in which carbonyl group is conjugated with an alkene, demonstrate strong antifungal activity. Such compounds include neral and geranial. 

### 2.4. Antifungal Activity of Artificial Blends

**Figure 1:**
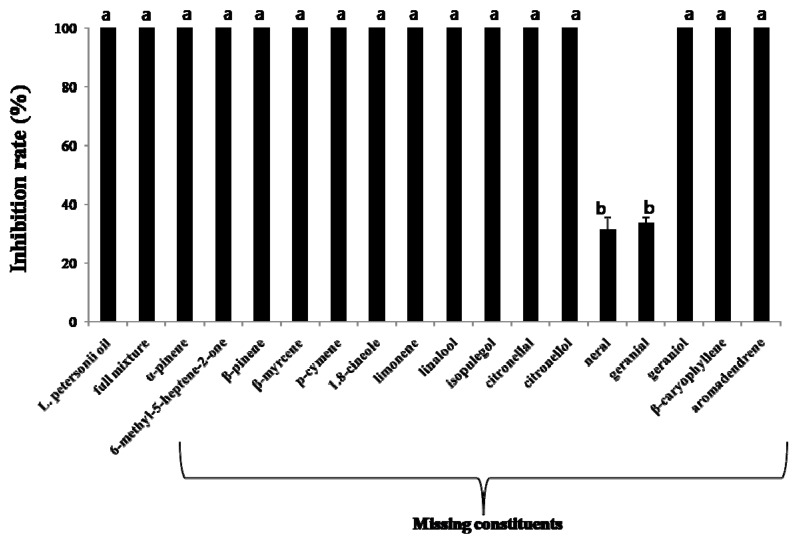
Fumigant antifungal activity of the oil, full mixture, and selected blends of the constituents of *L. petersonii* essential oil against *Aspergillus flavus*. The air concentration of *L. peterisonii* was 28 × 10^−3^ mg/mL. The air concentration of the full mixture of *L. peterisonii* oil was 25.43 × 10^−3^ mg/mL. The concentrations of other blends were determined by removing each constituent equivalent to the ratio identified in *L. peterisonii* oil. Mean values corresponding to each treatment with different letters are significantly different from each other (F_1__6,__51_ = 1.158, *p* < 0.0001, Scheffe’s test).

Elimination assays of *L. petersonii* oils indicated that the omission of neral and geranial from the artificial mixture resulted in a significant decrease in the fumigant antifungal activities of the blend (F_16,51_ = 1.158, *p* < 0.0001; Figure 1). These results indicate that neral and geranial are major contributors to the fumigant antifungal activity of *L. petersonii* oil and that they act synergistically with respect to their antifungal activity against *A. flavus*. Omission of other compounds from the artificial mixture did not cause a significant difference in the fumigant toxicity of the blend. Jiang *et al*. [36] have suggested that chemicals that protect plants against fungal infections are more effective when they contain compounds that act by different mechanisms.

## 3. Experimental

### 3.1. Fungal Strains and Culture Conditions

The three *Aspergillus* species (*Aspergillus ochraceus*, *A. niger*, and *A. flavus*) used in this study were supplied by KACC (Korean Agricultural Culture Collection, Suwon city, Korea). They were routinely maintained on malt extract agar (MEA) at 28 °C.

### 3.2. Essential Oils and Chemicals

Essential oils of *Eucalyptus citriodora*, *E. smithii*, *E. globulus*, *E. radiata*, *E. dives*, *E. polybractea*, *M. dissitiflora*, *M. quinquenervia*, *M. uncinata*, *M. linariifolia*, and *L. petersonii* were purchased from G.R. Davis Pty. Ltd. (Riverstone NSW 2765, Australia). Isopulegol (purity, 99%), 1,8-cineole (purity, 99%), (+)-limonene (purity, 97%), citronellol (purity, 95%), citronellal (purity, 85%) and myrcene (purity, 95%) were purchased from Sigma-Aldrich (Milwaukee, WI, USA). Geraniol (purity, 96%), aromadendrene (purity, 97%), and *p*-cymene (purity, 95%) were purchased from Fluka (Buchs, Switzerland). *α*-Pinene (purity, 95%), *β*-caryophyllene (purity, 90%), and *β*-pinene (purity, 94%) were purchased from Tokyo Kasei (Tokyo, Japan), and linalool (purity, 98%) was purchased from Wako Pure Chemical Industries Ltd. (Osaka, Japan). The synthesis of 6-methyl-5-hepten-2-one, geranial, and neral was performed according to our published method [15].

### 3.3. Chemical Analysis of *L. petersonii* Oil

The chemical analysis of *L. petersonii* plant essential oils was performed according to our published method [15].

Antifungal activities were determined following the methods of Lee *et al*. [15]. MEA (malt extract agar, Difco, Sparks, MD, USA) plates were prepared using plastic Petri dishes (87 mm). Each agar-mycelial plug (diameter, 7 mm) of *A. ochraceus*, *A. niger*, and *A. flavus* were inoculated into the center of the Petri dish. Lids were placed upside down on the Petri dishes. Essential oils or their constituents were introduced onto a paper disc (8 mm, Advantec, Tokyo, Japan), which was placed on the agar-free lid of the Petri dish. Petri dishes were sealed with parafilm to prevent the leaking of test oils and compounds. After inoculation, the plates were incubated at 28 °C in the dark. Colony diameter was measured after growth in the control treatment had completely covered the Petri dishes (average, 16–17 days for *A. ochraceus*, 14 days for *A. niger*, and 10–11 days for *A. flavus*). All treatments were replicated 4 times. The percent difference of growth inhibition between the treatment and control groups were calculated using the following formula:


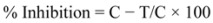


where C is an average of the four replicates of hyphal extension (mm) in control plates, and T is an average of 4 replicates of hyphal extension (mm) of plates treated with essential oils, an individual compound, and or artificial blends of *L. petersonii* constituents.

### 3.5. Antifungal Activity of Artificial Blends

To determine the contribution of each constituent of essential oil to the inhibition of growth of *A. flavus*, we prepared blends of all constituents for *L. petersonii* oil that mimicked the natural oil. We also prepared a number of blends, each lacking one constituent (Figure 1). Blends were based on the natural composition of the *L. petersonii* oil, as indicated by GC-FID (Table 2). The concentration of *L. petersonii* was 28 × 10^−3^ mg/mL. The concentration of the complete mixture of *L. peterisonii* oil was 25.43 × 10^−3^ mg/mL. The concentrations of other blends were determined by omitting each constituent equivalent to the ratio identified in the *L. petersonii* oil.

### 3.6. Stastistical Analysis

Mortality was converted to the arcsine square root values for analysis of variance (ANOVA). Treatment means were compared and contrasted by Scheffe’s test [37].

## 4. Conclusions

Our results indicate that the essential oil and certain of its constituents extracted from *L. petersonii* oil could be useful for inhibiting the growth of three *Aspergillus* species. However, further research is necessary for determining the practical applicability of *L. petersonii* oil and its constituents as novel fungicides, to determine its safety for humans, for developing formulations to improve its activity and stability, and to reduce treatment costs.
